# An Investigation on Spray-Granulated, Macroporous, Bioactive Glass Microspheres for a Controlled Drug Delivery System

**DOI:** 10.3390/ma14113112

**Published:** 2021-06-06

**Authors:** Henni Setia Ningsih, Liu-Gu Chen, Ren-Jei Chung, Yu-Jen Chou

**Affiliations:** 1 Department of Mechanical Engineering, National Taiwan University of Science and Technology, No. 43, Sec. 4, Keelung Rd., Taipei 10607, Taiwan; d10704805@mail.ntust.edu.tw; 2 Department of Engineering and System Science, National Tsing Hua University, No. 101, Sec. 2, Kuang-Fu Rd., Hsinchu 300044, Taiwan; liu31448@gmail.com; 3 Department of Chemical Engineering and Biotechnology, National Taipei University of Technology, No. 1, Sec. 3, Zhongxiao E. Rd., Taipei 10608, Taiwan; rjchung@ntut.edu.tw

**Keywords:** bioactive glass, spray granulation, electron microscopy, controlled drug delivery

## Abstract

Bioactive glass (BG) has been regarded as an excellent candidate for biomedical applications due to its superior properties of bioactivity, biocompatibility, osteoconductivity and biodegradability. Thus, in this study, we aimed to fabricate drug carriers that were capable of loading therapeutic antibiotics while promoting bone regeneration using macroporous BG microspheres, prepared by a spray drying method. Characterizations of particle morphology and specific surface area were carried out via scanning electron microscopy and nitrogen adsorption/desorption isotherm. Evaluations of in vitro bioactivity were performed based on Kokubo’s simulated body fluid to confirm the formation of the hydroxyapatite (HA) layer after immersion. In addition, the in vitro drug release behaviors were examined, using tetracycline as the therapeutic antibiotic in pH 7.4 and 5.0 environments. Finally, the results showed that BG microspheres of up to 33 μm could be mass-produced, targeting various therapeutic situations and their resulting bioactivities and drug release behaviors, and related properties were discussed.

## 1. Introduction

Bioactive glass (BG) has been considered a potential material for biomedical applications due to its remarkable properties such as non-toxicity, osteoconductivity, bioactivity, biocompatibility and biodegradability [1,2]. Since BG was first invented in 1971, various studies have demonstrated that the formation of hydroxyapatite (HA) layers could be found on the surface of BG when in contact with biological fluids [3,4,5]. These HA layers possess great osteogenic capability and are able to bond to human bones, which diminishes rejection and inflammation. Hence, BG has been widely used in field biomedical applications such as dermal filler [6], tooth filler [7] and radioisotope vectors [8]. In addition, BG has also been commonly used in clinical surgery [9] for repairing defects [10] in osseous [11] and lesion sites [12]. However, the implants have possibly been associated with osteomyelitis incidence, or the subsequent failure of implant, which has required additional surgery [13,14,15]. Meanwhile, the osteogenic capability of BG could not satisfy all clinical states [16]. Therefore, various studies have focused on introducing a drug delivery system into implant material in order to reduce the bacteriological risk associated with the use of antibiotic-loaded BG [17]. Numerous studies have reported the synthesis of drug-carrying particles possessing mesoporous structures to target different treatments [18,19,20].

In the past few decades, studies have demonstrated different preparation methods for BG. Among all these methods, the sol-gel method reported by Li et al. in 1991 has become the most popular process, owing to its friendly operation, controllable composition and low processing temperature (500–600 °C) [21]. The sol-gel method is capable of fabricating BG particles with different shapes and sizes with good osteogenic properties compared to traditional, melt-derived BG [22,23,24]. Although studies have demonstrated the preparation of BG particles with various mesoporous structures by the sol-gel or micro-emulsion methods, the particles still have had the issue of agglomeration, which reduces drug delivery capacity [25,26,27]. In addition, current synthetic methods for fabricating large-pore, mesoporous BG microspheres also encounter some difficulties, such as requiring a complex preparation process with the use of various organic pore forming agents that is relatively low-yield and time consuming and cannot satisfy mass production demands [28]. 

The spray drying method is a mature technique that has been used in the pharmaceutical field and has become one of the popular synthetic methods for BG fabrications in recent years. Studies have shown great promise for its high purity production at low calcination temperatures, and the products prepared by the spray drying technique have integrated benefits such as continuous process, mass production and size-controllable merit [29]. For instance, Chou et al. reported that by adjusting the processing parameters, the sphere sizes and morphologies of the BG microspheres could be optimized, targeting different applications [30]. In addition, the preparation of BG powders using the spray granulation method was reported, with controllable sphere sizes of up to 35 μm [31,32]. However, the specific surface areas of the reported granulated BG particles were too small to be considered for drug carrier applications. 

To overcome the above problem, the preparation of macroporous BG microspheres, in which poly (methyl methacrylate) (PMMA) was added as the hard template to increase the specific surface area, was carried out in this study. In addition, gelatin was used to encapsulate the BG specimens for further control of the drug release rate. Characterizations of particle morphologies and specific surface areas were examined using scanning electron microscopy (SEM) and the Brunauer–Emmett–Teller (BET) method. Finally, in vitro bioactivity was carried out following Kokubo’s protocol and evaluated using Fourier transform infrared spectroscopy (FTIR), while tetracycline hydrochloride (TC) and fluorescence microplate reader were employed for examination of the in vitro drug release profiles, under two treatment conditions of pH 7.4 and 5.0, to simulate the healthy and osteomyelitis conditions.

## 2. Materials and Methods

### 2.1. Synthesis

In this study, BG microspheres, based on 58S (60 mol.% SiO_2_, 35 mol.% CaO, and 5 mol.% P_2_O_5_), were synthesized using both spray drying and the spray granulation process. Firstly, spray-dried BG powders were fabricated, using tetraethyl orthosilicate (TEOS, >98.0 wt.%, Seedchem, Camberwell, Melbourne, Australia), calcium nitrate tetrahydrate (CaN, 98.5 wt.%, Showa, Gyoda, Saitama, Japan) and triethyl phosphate (TEP, 99.0 wt.%, Alfa Aesar, Haverhill, MA, USA) as the sources of SiO_2_, CaO and P_2_O_5_, and the precursor solutions were prepared by dissolving the above precursors (TEOS, CaN, and TEP) in 0.5M HCl and ethanol, based on a nominal ratio of 58S. Next, 1 L deionized water was added for dilution, and all precursor solutions were stirred at 25 °C for 4 h to achieve solution homogeneity. Following that, the solutions were disseminated into fine droplets using a high-speed spinning disc at 20,000 rpm. The fine droplets were then led into the spray dryer machine (SD D0-03, IDTA machinery, New Taipei City, Taiwan), wherein the chamber inlet/outlet temperature was set at 200/80 °C with atmospheric air, and a precursor flow rate of 20 mL/min was set to form the spray-dried powders. The resulting powders were obtained in an attached collection tube and were then calcined at 600 °C for 1 h to obtain the preliminary 58S powder. 

For the fabrication of macroporous BG microspheres, the precursor solutions were prepared by adding 20 wt.% spray dried BG powder, 1 wt.% poly (N-vivylactamide) ((C_4_H_7_NO)_n_, Showa, Gyoda, Saitama, Japan) as a dispersant, and various concentrations (0, 5, 10 and 20 wt.%) of PMMA as a hard template [33,34,35] into 40 mL deionized water and stirred at 25 °C for 4 h. Meanwhile, the binder solutions were prepared by dissolving 5 wt.% PVA ((C_2_H_4_O)_n_, ACROS) and 0.25 wt.% emulsified polyacrylic resin (Sun-Yarak technology) into 60 mL deionized water and stirred at 25 °C for 18 h. Next, by mixing both precursor and binder solutions, the mixtures were stirred at 25 °C for an additional 18 h. Finally, the macroporous BG microspheres were prepared following the spray drying procedure, as described above. The resulting powders were calcined at 800 °C for another 6 h to remove binders, dispersants and structural templates to avoid the influence of additives on bioactivity. 

Finally, a photosensitive drug, tetracycline hydrochloride (TC, C_22_H_24_N_2_O_8_∙HCl, Biosynth), was employed for the preparation of drug carriers. First, by dissolving TC into ethanol based on a concentration of 0.3 mg/mL, the macroporous BG microspheres were immersed into the TC solution with a solid to liquid ratio of 10 mg/mL. Next, the solution was removed, and the specimens were dried at 70 °C in an oven for 12 h to complete the loading process. Furthermore, the additional process was carried out by immersing the specimens into a gelatin solution (C_102_H_151_O_39_N_31_, Sigma-Aldrich, St. Louis, MO, USA) and drying at 70 °C for 6 h to form the gelatin-capsulated BG microspheres for controlled drug release.

### 2.2. Characterization

The surface morphologies of all macroporous BG microspheres were examined using a field emission SEM (Quanta 3D FEG, FEI, Hillsboro, OR, USA). The sphere size distributions and average sphere sizes were statistically calculated by selecting more than 300 particles from among several SEM images. In addition, the specific surface areas of the BG specimens were measured by the BET method from nitrogen adsorption and desorption isotherms. All BG specimens were degassed at 150 °C before measurement and were then placed on a constant volume adsorption apparatus (Novatouch LX2, Quantachrome Instruments, Boynton Beach, FL, USA). The isotherms were recorded at −196 °C, and the specific surface areas were computed.

The in vitro bioactivity of the macroporous BG microspheres was evaluated based on Kokubo’s protocol [36] by immersing the specimens into the simulated body fluid (SBF), with a ratio of 20 mg/mL. The test solutions were kept in an orbital shaker set at 37 °C, while the SBF was replaced once per day for 7 d. The immersed specimens were washed three times with both acetone and deionized water before drying at 70 °C for a day. Finally, SEM were used to observe the surfaces of the specimens, while FTIR (FTS-1000, Digilab, Hopkinton, MA, USA) spectra were collected from 1600 to 400 cm^−1^, examining the fingerprint regions of P-O vibrations for confirmation of HA formation.

Finally, for measurements of the in vitro drug release profile, all BG specimens were immersed in phosphate-buffered saline (PBS) solution at 37 °C for various durations of 0, 1, 2, 3, 4, 5, 6, 12, 24, 48, 72, 96 and 120 h. In addition, two solutions of pH 7.4 and 5.0 were used to simulate drug release behaviors in the healthy and osteomyelitis environments. Then, a fluorescence microplate reader (VarioskanTM FLASH, ThermoFisher, Waltham, MA, USA) was employed to measure the values of the optical density of the TC at a wavelength of 360 nm. Finally, drug release at each duration was measured three times, and drug release profiles were obtained based on the cumulative calculations.

## 3. Results

**Figure 1:**
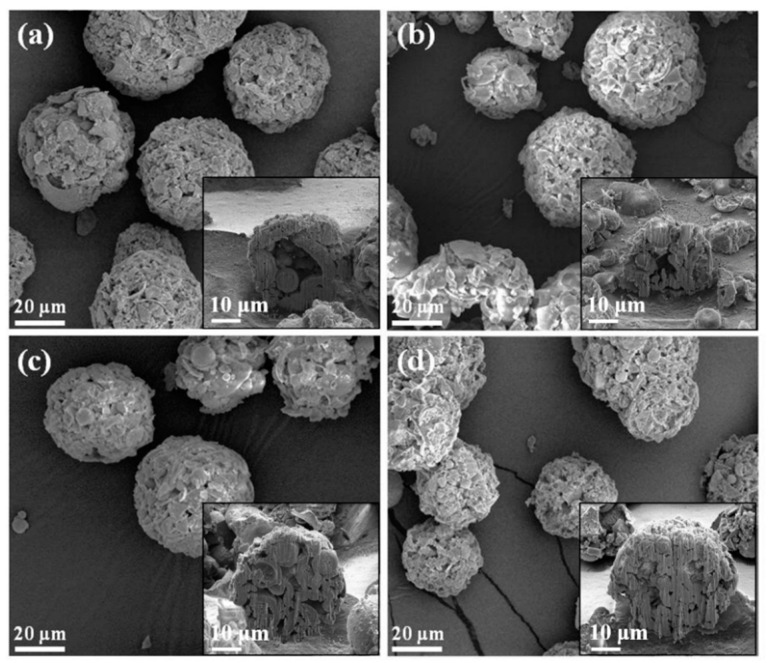
SEM images of BG microspheres treated with (**a**) 0, (**b**) 5, (**c**) 10 and (**d**) 20 wt.% PMMA, with insets of their cross-sectional images.

**Figure 2:**
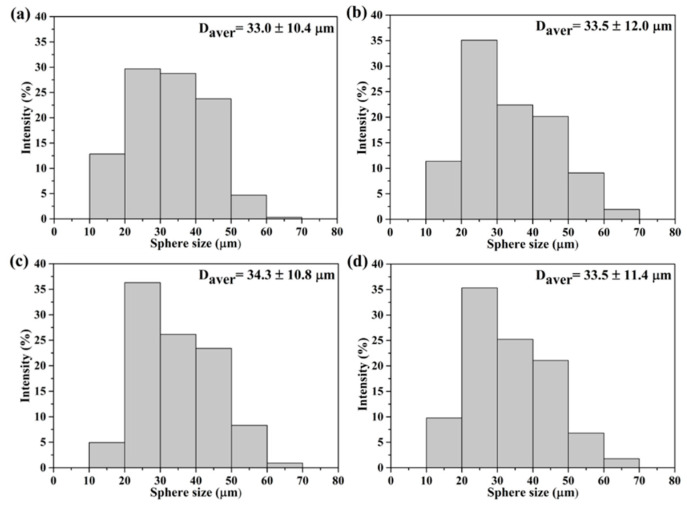
Histograms of sphere size distributions of (**a**) 0, (**b**) 5, (**c**) 10 and (**d**) 20 wt.% PMMA-treated BG microspheres.

The SEM images of all macroporous BG specimens treated with 0, 5, 10 and 20 wt.% PMMA are shown in Figure 1, with insets of their cross-sectional images. The results showed that a spherical morphology could be observed from all BG specimens. In addition, open pore structures were found within all specimens, while inner morphologies of macroporous could be observed. Meanwhile, the sphere sizes were statistically measured with more than 300 particles using numerous SEM images. The resulting histograms of sphere size distributions are shown in Figure 2, and all graphs show a normal distribution, with sphere sizes ranging from 10 to 70 μm. Moreover, the averaged sphere sizes were computed as 33.0 ± 10.4, 33.5 ± 12.0, 34.3 ± 10.8 and 33.5 ± 11.4 μm for the 0, 5, 10 and 20 wt.% PMMA-treated BG microspheres, respectively. Meanwhile, the specific surface areas were measured by BET, giving results of 44.0 ± 0.4, 57.5 ± 4.7, 72.0 ± 3.9 and 59.4 ± 1.6 m^2^/g for the BG specimens treated with 0, 5, 10 and 20 wt.% PMMA, respectively. In brief, the SEM results showed the independence of the sphere size as related to the PMMA concentration, while the variance of specific surface areas with PMMA additives was observed with the BET results.

**Figure 3:**
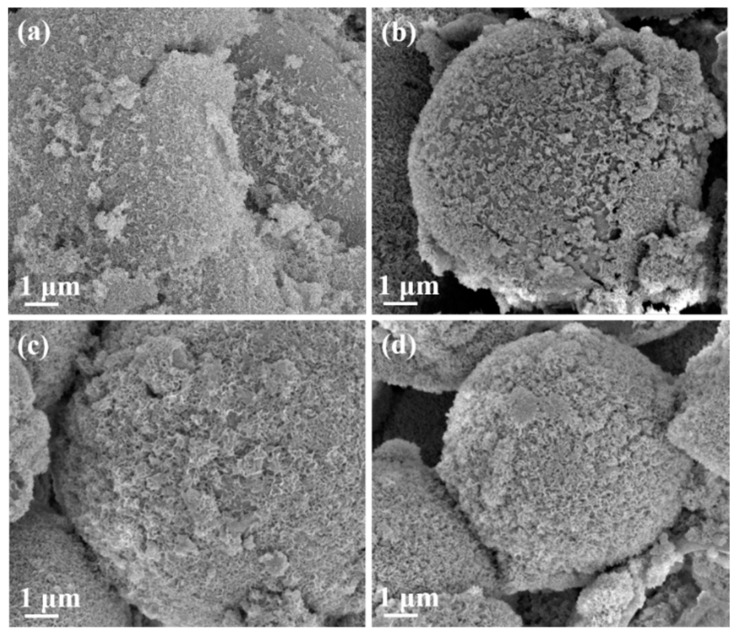
SEM images of BG microspheres treated with (**a**) 0, (**b**) 5, (**c**) 10 and (**d**) 20 wt.% PMMA after SBF immersion for 168 h.

**Figure 4:**
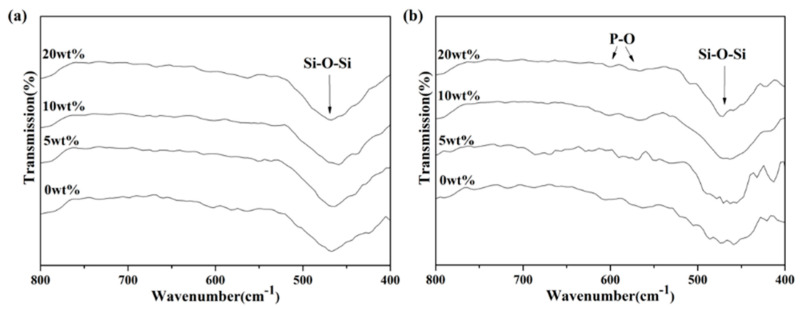
FTIR spectra of BG microspheres treated with 0, 5, 10 and 20 wt.% PMMA (**a**) before and (**b**) after SBF immersion for 168 h.

Evaluations of bioactivity were carried out via SEM and FTIR. Figure 3 shows the SEM images from all macroporous BG microspheres after SBF immersion for 24 h. It can be seen from the graph that the formation of needle-shaped HA was observed on the surface of each BG specimen. Meanwhile, the FTIR spectra were recorded within the range of 1400 to 400 cm^−1^, and the results are shown in Figure 4. Initially, Figure 4a shows the FTIR spectra of all BG specimens before SBF immersion. The resulting peaks at 1095, 802 and 482 cm^−1^ can be observed, which correspond to various Si–O–Si stretching and the bending bonds of the SiO_2_ tetrahedral structure [37,38]. In contrast, the FTIR spectra after SBF immersion are shown in Figure 4b. It could be seen from all spectra that, excluding the existing Si–O–Si peaks, additional peaks were observed at 566 and 598 cm^−1^ in all BG specimens after immersion into SBF. Both peaks corresponded to P-O vibration [39], which indicated HA formation. 

**Figure 5:**
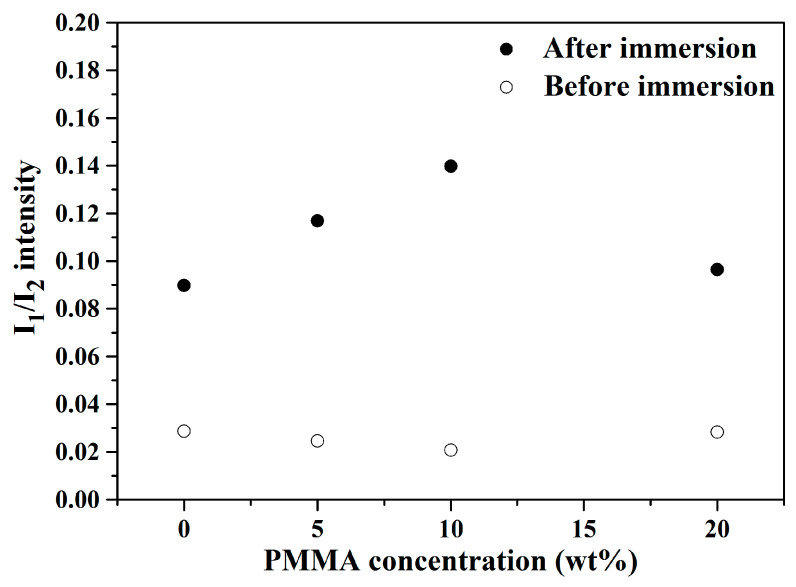
Computed I_1_/I_2_ intensity of 0, 5, 10 and 20 wt.% PMMA–treated BG microspheres before and after SBF immersion.

Moreover, the ratio of peak intensity (I_1_/I_2_) was computed following Li’s protocol for the quantification of bioactivity [21], wherein I_1_ was the P-O vibration (566 cm^−1^), while I_2_ was the Si–O–Si vibration (482 cm^−1^). The resulting I_1_/I_2_ values were calculated as 0.090, 0.117, 0.140 and 0.096 for the macroporous BG microspheres treated with 0, 5, 10 and 20 wt.% PMMA after immersion in SBF, respectively. Figure 5 shows the computed I_1_/I_2_ values of 0, 5, 10 and 20 wt.% PMMA-treated BG microspheres before and after immersion into SBF. It can be seen from the graph that the I_1_/I_2_ values increased significantly for all BG specimens after SBF immersion. Moreover, because higher I_1_/I_2_ values indicated higher bioactivity due to the formation of more hydroxyapatite, the results showed that the 10 wt.% PMMA-treated BG specimen had the best bioactivity among all BG specimens. In short, the bioactivity of all BG specimens could be confirmed via both SEM and FTIR results.

**Figure 6:**
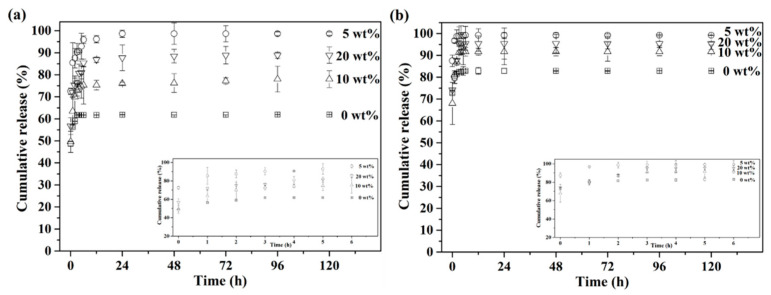
Cumulative tetracycline release profiles of 0, 5, 10 and 20 wt.% PMMA-treated BG microspheres in (**a**) pH 7.4 and (**b**) pH 5.0 PBS solution at 37 °C.

Furthermore, measurements of in vitro drug release were carried out under simulated healthy and osteomyelitis environments with pH 7.4 and 5.0. The cumulative drug release profiles of up to 120 h are presented in Figure 6, with insets of the initial release from 0 to 6 h. First, Figure 6a shows the drug release profile with pH 7.4, simulating the healthy environment. The results showed that the drug release profile stopped at 61% after 3 h for the BG specimens granulated with 0 wt.% PMMA, while the 5, 10 and 20 wt.% PMMA-treated BG specimens showed continuous drug releases up until 24 h at 99%, 76% and 88%, respectively. Meanwhile, Figure 6b shows the drug release profile, with pH 5.0 as the osteomyelitis environment. All drug release profiles stopped within 3 h for all BG specimens. In addition, faster drug release rates could be observed in an environment of pH 5.0 for all specimens as compared to pH 7.4, while the orders of cumulative drug release up until 120 h were the same in both conditions of pH 7.4 and 5.0, giving 5 wt.% PMMA-treated BG microspheres (○) > 20 wt.% PMMA-treated BG microspheres (▽) > 10 wt.% PMMA-treated BG microspheres (△) > 0 wt% PMMA-treated BG microspheres (□). 

**Figure 7:**
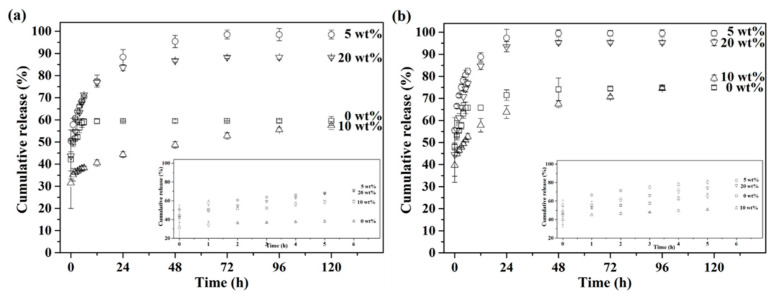
Cumulative tetracycline release profiles of gelatin-capsulated 0, 5, 10 and 20 wt.% PMMA-treated BG microspheres in (**a**) pH 7.4 and (**b**) pH 5.0 PBS solution at 37 °C.

For controlling and prolonging drug release behavior, gelatin encapsulation was employed for all drug-loaded BG microspheres. Their drug release profiles are shown in Figure 7. With gelatin encapsulation, both healthy and osteomyelitis environments were also simulated, with pH 7.4 and 5.0. The results are shown in Figure 7a,b. First, Figure 7a shows that the drug release behaviors of all gelatin-capsulated BG microspheres could be extended until 72 h in the condition of pH 7.4, showing a prolonged release profile as compared to non-capsulated specimens. In addition, for the pH 5.0 environment, as shown in Figure 7b, the drug release profiles of the gelatin-capsulated BG microspheres reached their maximum after releasing for 48 h. Moreover, similar trends of cumulative drug release until 120 h with non-capsulated specimens could be observed in both pH conditions, resulting in gelatin-capsulated, 5 wt.%, PMMA-treated BG microspheres (○) > gelatin-capsulated, 20 wt.% PMMA-treated BG microspheres (▽) > gelatin-capsulated, 10 wt.% PMMA-treated BG microspheres (△) ≈ gelatin-capsulated, 0 wt.% PMMA-treated BG microspheres (□). These results indicated that the drug release behavior could be prolonged once the BG specimens were encapsulated with gelatin, hence controlling the drug release profile.

## 4. Discussion

**Figure 8:**
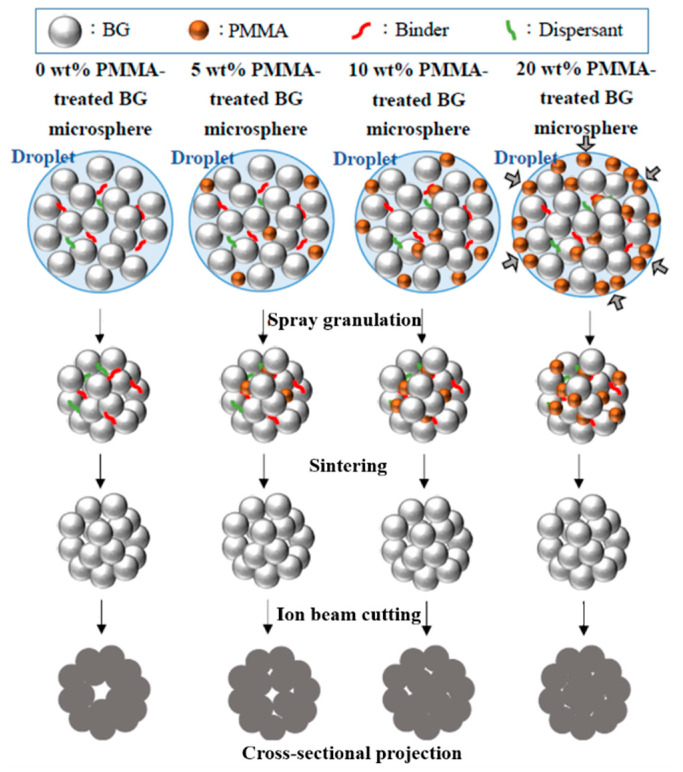
Schematic diagram of the formation mechanism of the PMMA-treated, granulated BG microspheres.

First, the formation mechanism of the macroporous BG microspheres should be discussed. To begin with, it should be noted that various parameters such as the selection of precursors, the concentration of solutions and the atomized droplet sizes might influence size and morphology during the spray granulation process. [30,40]. Based on the SEM images and sphere size distributions as shown in Figure 1 and Figure 2, the results showed that all macroporous BG microspheres treated with 0, 5, 10 and 20 wt.% PMMA exhibited a spherical morphology, while similar sphere size distributions and average sphere sizes of around 33 μm could be observed. Furthermore, the coefficient of variation was introduced, following the equation below, to identify the variation in all sphere sizes:

(1) $$\mathrm{Coefficient}\;\mathrm{of}\;\mathrm{Variation}=\frac{\mathrm{STD}}{D_{\mathrm{avg}}}\times 100\%$$

where the standard deviation is denoted as STD, while the average sphere size is denoted as D_avg_. The calculated coefficients of variation values were 31.5%, 35.7%, 31.5% and 34.0% for the 0, 5, 10 and 20 wt.% PMMA-treated BG microspheres, respectively. In addition, noticeable increases in the specific surface areas of PMMA-treated BG microspheres were 30.7%, 63.6% and 35.0% for 5, 10 and 20 wt.% PMMA-treated BG specimens as compared to the un-treated BG specimens, respectively, showing that the 10 wt.% PMMA-treated specimen had the highest specific surface area. This was owing to the hydrophobic and hydrophilic properties of the PMMA and BG particles. When the amount of PMMA reached a high volume, it became interconnected and preferred its segregation on the surface of the sphere. As a result, the amount of PMMA inside the spheres decreased, thus resulting in a lower porosity and consequently, a lower surface area after calcination. A proposed schematic diagram is shown in Figure 8. Therefore, smaller specific surface areas could be observed when an excessive amount of PMMA additive was used. In summary, the SEM images confirmed that all macroporous BG microspheres went through the typical spray granulation mechanism and resulted in consistent sphere size distributions. Meanwhile, the BET results showed that increased specific surface areas could be observed with the employment of PMMA as a hard template, indicating a successful preparation of macroporous, structure-targeting, drug-releasing applications.

Regarding the bioactivity of the macroporous BG microspheres, a needle-like morphology of the HA and P-O bending peak at 598 cm^−1^ has been reported by various studies [32,41], which is consistent with the SEM images and FTIR analysis shown in Figure 3 and Figure 4, confirming that all BG specimens are bioactive with HA formation once immersed in SBF for 7 d. In addition, the computed I_1_/I_2_ values indicated the order of bioactivity was 10 wt.% PMMA treated > 5 wt.% PMMA treated > 20 wt.% PMMA treated ≈ 0 wt.% PMMA treated, showing a good correlation with the order of specific surface areas [42]. 

Below, we discuss the in vitro drug release behaviors of all BG specimens. Based on the drug release profiles shown in Figure 6, the order of cumulative drug release up until 120 h was 5 wt.% > 20 wt.% > 10 wt.% > 0 wt % PMMA-treated BG microspheres, and this order was not in good agreement with the order of the measured surface areas. This might be owing to the fragmentation of BG microspheres during the drug loading process, which resulted in variations in surface area and led to different trends in drug release behavior. In addition, rapid drug release in the first hour could be observed from all non-capsulated BG specimens in both pH 7.4 and 5.0 conditions. Meanwhile, the drug release rate was much faster in pH 5.0 as compared to pH 7.4, giving complete drug releases within 3 h and 6 h in pH 5.0 and 7.4 environments. This was owing to the higher dissolution rate of TC in acidic conditions. However, in both cases, the drug release rate was too fast, which might cause dose dumping and result in low curing efficiency. Thus, the gelatin-capsulated BG microspheres were prepared to control the drug release rate, and the results are shown in Figure 7. In contrary to the non-capsulated BG microspheres, the drug release rates of the gelatin-capsulated BG microspheres decelerated significantly in both pH 7.4 and 5.0 environments. This resulted in extended drug release profiles of up to 72 h and 48 h in environments of pH 7.4 and pH 5.0. Because the gelatin used in this study was an acid-treated collagen with an isoelectric point between pH 6.0 to pH 9.0, there was no mutual repulsion within the PBS solution at pH 7.4 when gelatin-capsulated BG microspheres were immersed. Thus, the dissolved gelatin existed in the form of zwitterions, and the intermolecular forces were weakened, resulting in a decrease in solubility. Hence, a lower cumulative drug release of gelatin-capsulated BG microspheres was observed at pH 7.4 as compared to pH 5.0. Additionally, ANOVA analyses were performed against various parameters for various durations and shown in Table 1. The results showed that the *f*-Values for gelatin addition at 6 h and 12 h were 32.76 and 22.23, which were larger than the significant level at 5% (4.96 and 3.71), which indicated that it had a distinct influence on drug release behavior. However, the *f*-values decreased to 3.68 and 2.89 at 72 h and 120 h, lower than the significant level, thus showing that the effects of gelatin addition were not significant after 12 h. In contrary, the *f*-values for BET were 8.68, 11.55, 14.77 and 17.16 for pH values of 8.98, 9.91, 7.92 and 11.24 at 6 h, 12 h, 72 h and 120 h, respectively. All values were larger than the significant level, indicating that both variances of BET and pH were statistically significant in their drug release behavior at all times. 

Finally, to target various situations, the gelatin-capsulated 10 wt.% PMMA-treated BG specimens showed continuous drug release within 120 h, while the gelatin-capsulated 5 wt.% PMMA-treated BG specimens showed the fastest drug release rate within 48 h and had a cumulative release of 99.5%. This indicated that drug release behavior could be controlled to target different therapy situations. For instance, in an acute osteomyelitis situation, the 5 wt.% PMMA-treated BG specimen could provide a rapid and massive drug release behavior to support instant therapeutic activity, while the 10 wt.% PMMA-treated BG specimen could be suitable for preventing bacterial infections, owing to its slow and prolonged drug release behavior at pH 7.4.

## 5. Conclusions

In this study, successful fabrications of macroporous BG microspheres via a spray drying method were demonstrated. Based on the results of SEM and their corresponding sphere size distributions, all BG specimens followed a typical spray-dried formation mechanism, which exhibited consistent morphology, independent of the PMMA addition. Meanwhile, the bioactivity tests were evaluated, and all BG specimens were confirmed to be bioactive, with the formation of hydroxyapatite once immersed into the SBF. Moreover, the drug release behaviors of both as-prepared and gelatin-capsulated BG specimens were examined, simulating healthy and osteomyelitis environments at pH 7.4 and 5.0, and their corresponding drug release profiles were discussed. Finally, it is believed that gelatin-capsulated BG specimens with controlled, therapeutic antibiotic release rates could be regarded as potential candidates in applications of drug carriers and other related practices.

## Figures and Tables

**Table 1:** ANOVA analysis for drug release behaviors against various parameters.

Source	D.F.	*f*-Value			
		6 h	12 h	72 h	120 h
Gelatin addition	1	32.76 *	22.23 *	3.68	2.89
BET	3	8.68 *	11.55 *	14.77 *	17.16 *
pH	1	8.98 *	9.91 *	7.92 *	11.24 *

* Significance level at 5%: F_1, 10_ = 4.96; F_3, 10_ = 3.71.

## Data Availability

Not applicable.

## References

[1] Hench L.L., Polak J.M. (2002). Third-generation biomedical materials. Science.

[2] Jones J.R. (2013). Review of bioactive glass: From Hench to hybrids. Acta Biomater..

[3] Hench L.L., Splinter R.J., Allen W.C., Greenlee T.K. (1971). Bonding mechanisms at the interface of ceramic prosthetic materials. J. Biomed. Mater. Res..

[4] Hench L.L. (1991). Bioceramics: From Concept to Clinic. J. Am. Ceram. Soc..

[5] Cao W., Hench L.L. (1996). Bioactive materials. Ceram. Int..

[6] Fiume E., Barberi J., Verné E., Baino F. (2018). Bioactive glasses: From parent 45s5 composition to scaffold-assisted tissue-healing therapies. J. Funct. Biomater..

[7] Kirsten A., Hausmann A., Weber M., Fischer J. (2015). Bioactive and Thermally Compatible Glass Coating on Zirconia Dental Implants. J. Dent. Res..

[8] Tilocca A. (2015). Realistic Models of Bioactive Glass Radioisotope Vectors in Practical Conditions: Structural Effects of Ion Exchange. J. Phys. Chem. C.

[9] Salinas A.J., Shruti S., Malavasi G., Menabue L., Vallet-Regí M. (2011). Substitutions of cerium, gallium and zinc in ordered meso-porous bioactive glasses. Acta Biomater..

[10] Profeta A.C., Prucher G.M. (2015). Bioactive-glass in periodontal surgery and implant dentistry. Dent. Mater. J..

[11] Hench L.L. (1998). Biomaterials: A forecast for the future. Biomater..

[12] Profeta A., Huppa C. (2016). Bioactive-glass in Oral and Maxillofacial Surgery. Craniomaxillofac. Trauma Reconstr..

[13] Rao N., Ziran B.H., Lipsky B.A. (2011). Treating osteomyelitis: Antibiotics and surgery. Plast. Reconstr. Surg..

[14] Nandi S.K., Bandyopadhyay S., Das P., Samanta I., Mukherjee P., Roy S., Kundu B. (2016). Understanding osteomyelitis and its treatment through local drug delivery system. Biotechnol. Adv..

[15] Papakostidis C., Kanakaris N.K., Pretel J., Faour O., Morell D.J., Giannoudis P.V. (2011). Prevalence of complications of open tibial shaft fractures stratified as per the gustilo-anderson classification. Injury.

[16] Fenton O.S., Olafson K.N., Pillai P.S., Mitchell M., Langer R. (2018). Advances in Biomaterials for Drug Delivery. Adv. Mater..

[17] Tabia Z., el Mabrouk K., Bricha M., Nouneh K. (2019). Mesoporous bioactive glass nanoparticles doped with magnesium: Drug de-livery and acellular in vitro bioactivity. RSC Adv..

[18] Hao N., Jayawardana K.W., Chen X., Yan M. (2015). One-step synthesis of amine-functionalized hollow mesoporous silica nano-particles as efficient antibacterial and anticancer materials. ACS Appl. Mater. Interfaces.

[19] Prokopowicz M., Czarnobaj K., Szewczyk A., Sawicki W. (2016). Preparation and in vitro characterisation of bioactive mesoporous silica microparticles for drug delivery applications. Mater. Sci. Eng. C.

[20] Wu C., Chang J. (2012). Mesoporous bioactive glasses: Structure characteristics, drug/growth factor delivery and bone regeneration application. Interface Focus.

[21] Li R., Clark A.E., Hench L.L. (1991). An investigation of bioactive glass powders by sol-gel processing. J. Appl. Biomater..

[22] Hu Q., Li Y., Miao G., Zhao N., Chen X. (2014). Size control and biological properties of monodispersed mesoporous bioactive glass sub-micron spheres. RSC Adv..

[23] Hench L.L., Wheeler D.L., Greenspan D.C. (1998). Molecular Control of Bioactivity in Sol-Gel Glasses. J. Sol-Gel Sci. Technol..

[24] Wang Y., Chen X. (2017). Facile synthesis of hollow mesoporous bioactive glasses with tunable shell thickness and good monodis-persity by micro-emulsion method. Mater. Lett..

[25] Chen S.-Y., Chou P.-F., Chan W.-K., Lin H.-M. (2017). Preparation and characterization of mesoporous bioactive glass from agri-cultural waste rice husk for targeted anticancer drug delivery. Ceram. Int..

[26] Tang J., Chen X., Dong Y., Fu X., Hu Q. (2017). Facile synthesis of mesoporous bioactive glass nanospheres with large mesopore via biphase delamination method. Mater. Lett..

[27] Wang X., Li W. (2016). Biodegradable mesoporous bioactive glass nanospheres for drug delivery and bone tissue regeneration. Nanotechnology.

[28] Shih C., Chen H., Huang L., Lu P., Chang H., Chang I. (2010). Synthesis and in vitro bioactivity of mesoporous bioactive glass scaffolds. Mater. Sci. Eng. C.

[29] Molino G., Bari A., Baino F., Fiorilli S., Vitale-Brovarone C. (2017). Electrophoretic deposition of spray-dried Sr-containing mesoporous bioactive glass spheres on glass–ceramic scaffolds for bone tissue regeneration. J. Mater. Sci..

[30] Chou Y.-J., Hsiao C.-W., Tsou N.-T., Wu M.-H., Shih S.-J. (2018). Preparation and in Vitro Bioactivity of Micron-sized Bioactive Glass Particles Using Spray Drying Method. Appl. Sci..

[31] Chandrasekaran A., Novajra G., Carmagnola I., Gentile P., Fiorilli S., Miola M., Boregowda M., Dakshanamoorthy A., Ciardelli G., Vitale-Brovarone C. (2016). Physico-chemical and biological studies on three-dimensional porous silk/spray-dried mesoporous bioactive glass scaffolds. Ceram. Int..

[32] Chen L., Huang Y., Chou Y. (2021). Preparation and characterization of spray-dried granulated bioactive glass micron spheres. Int. J. Appl. Ceram. Technol..

[33] Yang Z., Zhang Y., Schnepp Z. (2015). Soft and hard templating of graphitic carbon nitride. J. Mater. Chem. A.

[34] Yan X., Yu C., Zhou X., Tang J., Zhao D. (2004). Highly Ordered Mesoporous Bioactive Glasses with Superior In Vitro Bone-Forming Bioactivities. Angew. Chem. Int. Ed..

[35] Ma Y., Qi L. (2009). Solution-phase synthesis of inorganic hollow structures by templating strategies. J. Colloid Interface Sci..

[36] Kokubo T., Ito S., Huang Z.T., Hayashi T., Sakka S., Kitsugi T., Yamamuro T. (1990). Ca, P-rich layer formed on high-strength bioactive glass-ceramic A-W. J. Biomed. Mater. Res..

[37] Innocenzi P. (2003). Infrared spectroscopy of sol–gel derived silica-based films: A spectra-microstructure overview. J. Non-Cryst. Solids.

[38] Charoensuk T., Sirisathitkul C., Boonyang U., Macha I.J., Santos J., Grossin D., Ben-Nissan B. (2016). In vitro bioactivity and stem cells attachment of three-dimensionally ordered macroporous bioactive glass incorporating iron oxides. J. Non-Cryst. Solids.

[39] Fowler B.O. (1974). Infrared studies of apatites. I. Vibrational assignments for calcium, strontium, and barium hydroxyapatites utilizing isotopic substitution. Inorg. Chem..

[40] Messing G.L., Zhang S.-C., Jayanthi G.V. (1993). Ceramic Powder Synthesis by Spray Pyrolysis. J. Am. Ceram. Soc..

[41] Tseng C.-F., Fei Y.-C., Chou Y.-J. (2020). Investigation of in vitro bioactivity and antibacterial activity of manganese-doped spray pyrolyzed bioactive glasses. J. Non-Cryst. Solids.

[42] Hong B.-J., Shih S.-J. (2017). Novel pore-forming agent to prepare of mesoporous bioactive glass using one-step spray pyrolysis. Ceram. Int..

